# The matrix-forming phenotype of cultured human meniscus cells is enhanced after culture with fibroblast growth factor 2 and is further stimulated by hypoxia

**DOI:** 10.1186/ar1929

**Published:** 2006-03-17

**Authors:** Adetola B Adesida, Lisa M Grady, Wasim S Khan, Timothy E Hardingham

**Affiliations:** 1 UK Centre for Tissue Engineering at The Wellcome Trust Centre for Cell-Matrix Research, Faculty of Life Sciences, Michael Smith Building, The University of Manchester, Manchester, Oxford Road, M13 9PT, UK

## Abstract

Human meniscus cells have a predominantly fibrogenic pattern of gene expression, but like chondrocytes they proliferate in monolayer culture and lose the expression of type II collagen. We have investigated the potential of human meniscus cells, which were expanded with or without fibroblast growth factor 2 (FGF2), to produce matrix in three-dimensional cell aggregate cultures with a chondrogenic medium at low (5%) and normal (20%) oxygen tension. The presence of FGF2 during the expansion of meniscus cells enhanced the re-expression of type II collagen 200-fold in subsequent three-dimensional cell aggregate cultures. This was increased further (400-fold) by culture in 5% oxygen. Cell aggregates of FGF2-expanded meniscus cells accumulated more proteoglycan (total glycosaminoglycan) over 14 days and deposited a collagen II-rich matrix. The gene expression of matrix-associated proteoglycans (biglycan and fibromodulin) was also increased by FGF2 and hypoxia. Meniscus cells after expansion in monolayer can therefore respond to chondrogenic signals, and this is enhanced by FGF2 during expansion and low oxygen tension during aggregate cultures.

## Introduction

The meniscus is a fibrocartilaginous tissue found within the knee joint; it is responsible for shock absorption, load distribution, joint stability and protection of the articular cartilage [1-3]. Unfortunately, the reparative ability of the meniscus is limited, and injuries to the tissue are often treated by partial or total menisectomy, which is known to be associated with detrimental changes in joint function and high incidence of early osteoarthritis [4,5]. Cell-based tissue engineering strategies have been proposed for the generation of meniscus substitute to aid repair of the tissue [6-10]. Like most musculoskeletal tissues, the biomechanical and functional properties of the meniscus depend on the composition and organization of its extracellular matrix (ECM) [1,11]. The cells that produce and maintain this ECM have been called fibrochondrocytes [12]. Although the predominant morphology of these cells resembles that of the chondrocytes of articular cartilage [1], they produce predominantly type I collagen with smaller amounts of types II, III, V and VI collagens, and small amounts of aggrecan [13]. Isolated primary human meniscus cells show some characteristics similar to those of chondrocytes because during expansion in monolayer culture there is a sharp decrease in the expression of collagen type II and a change to predominantly fibroblast-like morphology [7]. This decline in type II collagen expression is reminiscent of the loss of differentiated phenotype of articular chondrocytes, and the use of these cells for tissue regeneration of meniscus might lead to the production of ECM of inferior biomechanical properties. Several investigators have reported strategies to restore the matrix-forming phenotype of articular chondrocytes. These include culturing chondrocytes at high cell densities to prevent cell flattening [14], in alginate gels [15] to retain the round chondrocytic morphology, in liquid suspension or in the presence of actin-disrupting agents, in the presence of fibroblast growth factor 2 (FGF2) [16], retroviral transduction with Sry-related high-mobility group (HMG) box-9 (SOX9) [17], in three-dimensional (3D) cell aggregate cultures with chondrogenic stimuli [18] and low oxygen tension (mild hypoxia) [19-21].

In the present study we have investigated the presence of chondrogenic growth factors and hypoxia with human meniscus cells expanded in monolayer culture to determine their chondrogenic potential. The effect of FGF2 on chondrogenic potential of meniscus cells was particularly of interest, because it had been shown to stimulate the growth [22] of meniscus cells *in vitro *and also to maintain the ability of monolayer expanded chondrocytes to redifferentiate [16,23].

## Materials and methods

### Cell isolation and expansion

With informed consent, full-thickness meniscus was harvested aseptically from the tibial plateau of patients (aged 48 to 69 years) undergoing total knee replacements. Meniscus cells were released by incubation for 16 hours at 37°C in type II collagenase (0.2% w/v) in a standard medium, DMEM supplemented with 10% FCS, 100 units/ml penicillin and 100 units/ml streptomycin (all from Cambrex, Wokingham, UK), with l-glutamine (2 mM). Isolated cells were plated at 10^4 ^cells/cm^2 ^and cultured in standard medium with or without FGF2 (5 ng/ml) (human recombinant; R&D systems, Abingdon, UK (added after overnight cell adherence) at 37°C and 20% O_2_. After about 2 weeks, when cells were subconfluent, first-passage (P1) cells were detached with trypsin-EDTA (Invitrogen, Paisley, Renfrewshire, UK) and split at a 1:2 ratio; culture was continued to produce second-passage (P2) cells, which were used for experiments. Doubling times of P1 and P2 human meniscus cells were obtained by plating P1 and P2 meniscus cells at 5 × 10^5 ^cells in 75 cm^2 ^tissue culture flasks in the presence and absence of FGF2 (5 ng/ml). Meniscus cell number was evaluated at regular timed intervals in quadruplicate by cell counting after treatment with trypsin. The doubling time of a cell population during the exponential growth phase was calculated as the slope of *T *against ln *N*/*N*_0_), where *N*_0 _and *N *are the cell number at the beginning and end of the exponential growth time (*T*), respectively [16].

Three-dimensional cell aggregate cultures of meniscus cells were formed by the centrifugation of 5 × 10^5 ^cells in 15 ml conical culture tubes (Corning, Loughborough, UK) at 1,200 r.p.m. for 5 minutes. The cell aggregates were cultured in 0.5 ml of DMEM supplemented with chondrogenic factors, namely ITS+1, 1.0 mg/ml insulin from bovine pancreas, 0.55 mg/ml human transferrin (substantially iron-free), 0.5 μg/ml sodium selenite, 50 mg/ml bovine serum albumin and 470 μg/ml linoleic acid 10 nM dexamethasone, 10 ng/ml transforming growth factor β_3_, 25 μg/ml ascorbate 2-phosphate (all from Sigma, Poole, UK) with 10% FCS for 14 days with 5% CO_2 _under normal oxygen (20% O_2_) or low oxygen tension (5% O_2_) at 37°C. At the end of the culture period, the wet weights of cell aggregates were recorded. Control monolayer cultures of meniscus cells with or without FGF2 expansion (R&D systems) were set up in six-well plates at a 10^5 ^cells per well in standard medium. Monolayer controls were similarly cultured for 14 days under normoxic and hypoxic culture conditions, with standard medium change every 2 days.

### Gene expression analysis

Total RNA was prepared from whole tissue, monolayer cells and cell aggregate cultures with the use of Tri-Reagent (Sigma). Total RNA from tissue was isolated after homogenization with a Braun Mikrodismembranator. Cell aggregate cultures were ground up in the Tri-Reagent with Molecular Grinding Resin (Geno Technology Inc., St Louis, MO, USA). To minimize any changes in gene expression, cultures caps were closed before removal from the low-oxygen incubator, and cell aggregates and monolayers were immediately (less than 1 minute) transferred into Tri-reagent. cDNA was synthesised from 10 to 100 ng of total RNA with the use of global amplification methodology [24]. Globally amplified cDNAs were diluted 1:1000 and 1 μl aliquots of the diluted cDNA were amplified by polymerase chain reaction in a 25 μl reaction volume in an MJ Research Opticon 2 real-time thermocycler with a SYBR Green Core Kit (Eurogentec, Seraing, Belgium), with gene-specific primers designed by using ABI Primer Express software. Relative expression levels were normalised with β-actin and calculated with the use of the 2^-Δ*Ct *^method [25]. All primers were from Invitrogen. All primer sequences were designed on the basis of human sequences as follows: aggrecan, 5'-AGGGCGAGTGGAATGATGTT-3' (forward) and 5'-GGTGGCTGTGCCCTTTTTAC (reverse); β-actin, 5'-AAGCCACCCCACTTCTCTCTAA-3' (forward) and 5'-AATGCTATCACCTCCCCTGTGT-3' (reverse); biglycan, 5'-TTGCCCCCAAACCTGTACTG-3' (forward) and 5'-AAAACCGGTGTCTGGGACTCT-3' (reverse); COL1A2, (collagen) 5'-TTGCCCAAAGTTGTCCTCTTCT-3' (forward) and 5'-AGCTTCTGTGGAACCATGGAA-3' (reverse); COL2A1, 5'-CTGCAAAATAAAATCTCGGTGTTCT-3' (forward) and 5'-GGGCATTTGACTCACACCAGT-3' (reverse); COL3A1, 5'-GGCATGCCACAGGGATTCT-3' (forward) and 5'-GCAGCCCCATAATTTGGTTTT-3' (reverse); decorin, 5'-CAAGCTTAATTGTTAATATTCCCTAAACAC-3' (forward) and 5'-ATTTTATGAAGGGAGAAGACATTGGTTTGTTGACA-3' (reverse); fibromodulin, 5'-TGAAGCACCTTCCCTGAGAAG-3' (forward) and 5'-GGTTTGGCTTTTGTGGATTCC-3' (reverse); Sry-related HMG box-5 (long form), L-SOX5 5'-GAATGTGATGGGACTGCTTATGTAGA-3' (forward) and 5'-GCATTTATTTGTACAGGCCCTACAA-3' (reverse); Sry-related HMG box-6 (SOX6), 5'-CACCAGATATCGACAGAGTGGTCTT-3' (forward) and 5'-CAGGGTTAAAGGCAAAGGGATAA-3' (reverse); SOX9, 5'-CTTTGGTTTGTGTTCGTGTTTTG-3' (forward) and 5'-AGAGAAAGAAAAAGGGAAAGGTAAGTTT-3' (reverse).

### Biochemical analysis of cell aggregate cultures

After culture, cell aggregates were digested overnight in 20 μl of 10 units/ml papain (Sigma), 0.1 M sodium acetate, 2.4 mM EDTA, 5 mM l-cysteine, pH 5.8, at 60°C. The DNA content of the papain digest was determined by measuring Hoechst 33258 dye (Sigma) binding with a Hoeffer Dyna Quant 200 fluorometer. Glycosaminoglycans were assayed in the papain digest by using 1,9-dimethylmethylene blue (Aldrich, Poole, UK) [26,27] with shark chondroitin sulphate (Sigma) as standard.

### Histology and immunohistochemistry

Cell aggregates were fixed in 4% formaldehyde and embedded in paraffin wax; 5 μm sections were cut and stained with 0.1% safranin-O. For immunohistochemical analysis, sections were digested with chondroitinase ABC and then incubated with antibodies against collagen I (sc-8786) or collagen II (sc-7764) from Santa Cruz Biotechnology (Santa Cruz, CA, USA). Immunolocalised antigens were visualised with a biotin-conjugated donkey anti-goat secondary antibody (Santa Cruz Biotechnology) and a streptavidin-horseradish peroxidase or anti-rabbit horseradish peroxidase (Sigma) labelling kit with 3,3'-diaminobenzidine (Dako, Ely, UK). Images were captured with an Axioplan 2 (Carl Zeiss Ltd, Welwyn Garden City, UK) microscope and an AxioCam HRc camera (Carl Zeiss), with AxioVision 4.3 software (Carl Zeiss).

### Statistical analysis

Experiments were repeated in triplicate with cells from the same donor. Gene expression data are presented as the mean ± SD (standard deviation) for the replicates. Statistical significance differences between the gene expression values of normoxia and hypoxia cell aggregate cultures were determined with Student's unpaired *t *test.

## Results

### Cell population doubling during monolayer expansion of meniscus cells

**Figure 1:**
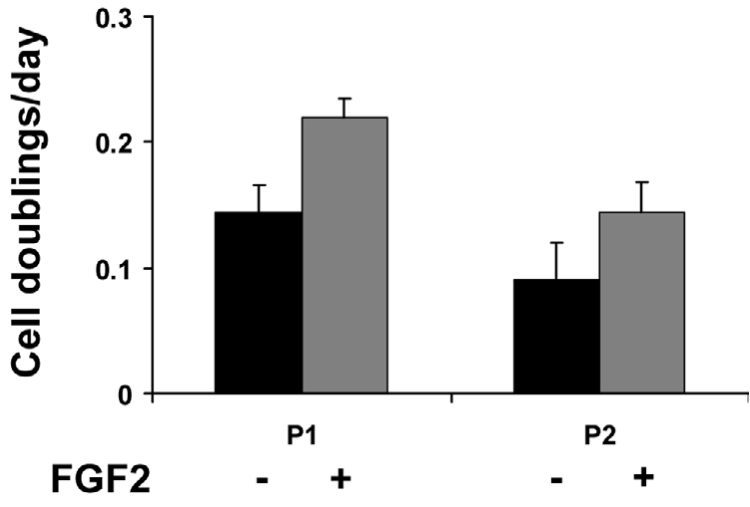
Cell doubling for P1 and P2 meniscus cells in the presence and absence of FGF2. Results are expressed as means ± SD (*n *= 4). FGF2, fibroblast growth factor 2; P1, passage 1; P2, passage 2.

Cells were isolated from human knee meniscus tissue and cultured in monolayer in the presence and absence of FGF2. The cells proliferated well in standard medium (without FGF2) and appeared fibroblastic with a flattened morphology. In the absence of FGF2, the rate of population cell doubling was 0.14 ± 0.02 per day at P1 and 0.09 ± 0.03 per day at P2. However, the cells that were expanded in the presence of FGF2 had an elongated spindle-like cell morphology and proliferated faster, with rates of population doubling 0.22 ± 0.02 per day at P1 and 0.14 ± 0.02 per day at P2 (Figure 1). The effect of FGF2 expansion on the chondrogenic potential of meniscus cells was investigated in 3D cell aggregates in the presence of growth factors known to promote matrix formation. In addition, the effect of low oxygen tension, which has previously been shown to enhance matrix assembly by chondrocytes, was investigated.

### Effect of FGF2 expansion on chondrogenic response of human meniscus cells

**Figure 2:**
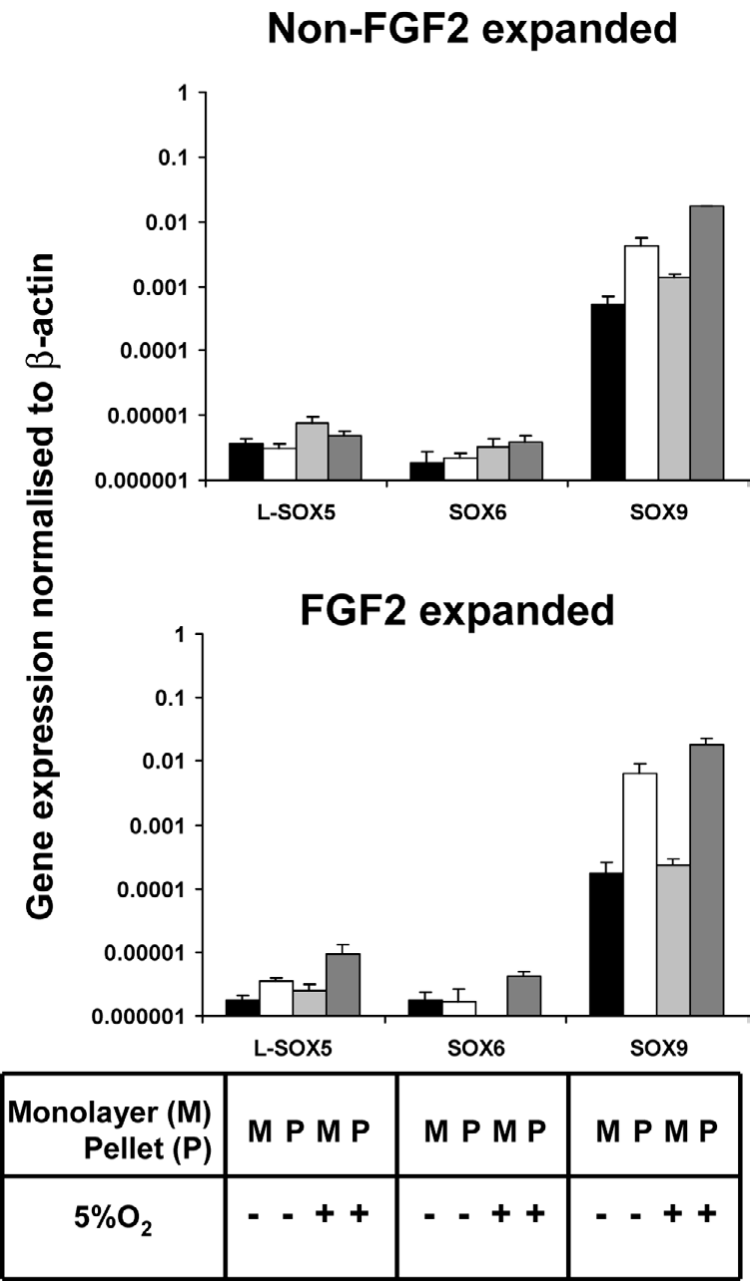
Sox gene expression in monolayer and cell aggregate cultures. Gene expression for monolayer cells (black bars) between P2 and P3 in standard medium (*n *= 3), for cell aggregate (white open bars) culture in chondrogenic differentiation medium after 14 days of culture under normoxia (*n *= 3). Gene expression for monolayer cells (light grey bars) between P2 and P3 in standard medium (*n *= 3) and for cell aggregate (dark grey bars) in chondrogenic differentiation medium after 14 days of culture under low oxygen tension (*n *= 3). *p < 0.05; **p < 0.001 (Student's unpaired *t *test) in cell aggregates from fibroblast growth factor 2 (FGF2)-expanded versus non-FGF2-expanded cells. P2, passage 2; P3, passage 3.

**Figure 3:**
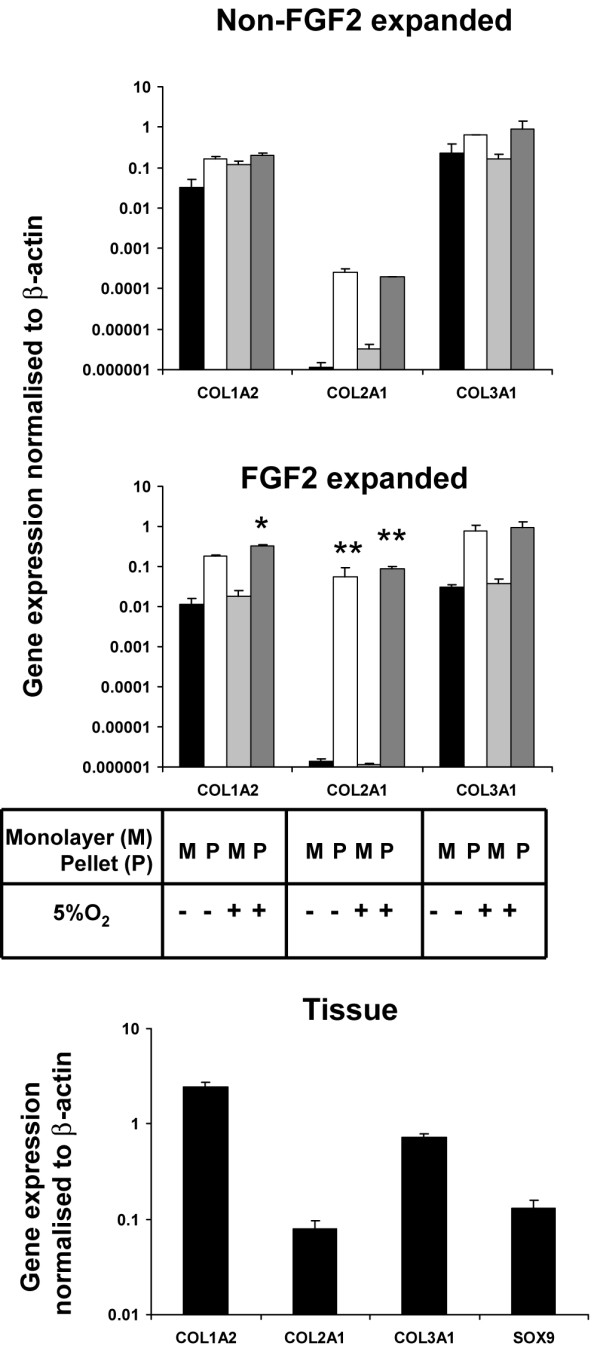
Collagen expression in monolayer and cell aggregate cultures, and in meniscus tissue with SOX9 expression (*n *= 3). Gene expression levels for monolayer cells (light grey bars) between P2 and P3 in standard medium (*n *= 3) and for cell aggregate (dark grey bars) cultures in chondrogenic differentiation medium (*n *= 3) under low oxygen tension after 14 days in culture. *p < 0.05; **p < 0.001 (Student's unpaired *t *test) in cell aggregates from fibroblast growth factor 2 (FGF2)-expanded versus non-FGF2-expanded cells.

One effect of the expansion with FGF2 on the gene expression of meniscus cells in monolayer culture was to suppress COL2A1 and SOX9 expression further than in non-FGF2-expanded cells. All expanded meniscus cells showed a major increase in the expression of COL2A1 after 14 days in cell aggregate cultures. There was a 236-fold increase in COL2A1 and an 8-fold increase in SOX9 in non-FGF2-expanded cells, but in FGF2-expanded cells the increase in COL2A1 was much higher (40,000-fold), as it was for SOX9 (35-fold) (Figures 2 and 3). FGF2 cells therefore showed a greater potential to regain COL2A1 and SOX9 expression and final levels of expression exceeded those of non-FGF2-expanded cells. The expression of COL2A1 and SOX9 was modulated further by culture at low oxygen tension. In non-FGF2-expanded cells COL2A1 increased 60-fold and SOX9 12-fold, whereas COL2A1 increased 80,000-fold and SOX9 80-fold in FGF2-expanded cells (Figures 2 and 3). However, the 80,000-fold increase in COL2A1 expression partly reflected the initial suppression of COL2A1 expression in FGF2-expanded monolayer cells. The effect of cell culture with FGF2 was clearly to generate a cell population that responded more positively to the cell aggregate culture conditions, and this response was further enhanced by culture at low oxygen tension. Surprisingly, the increase in COL2A1 expression was not accompanied by a significantly higher level of SOX9 expression (*p *> 0.05). The net effect of FGF2 expansion was therefore to have no significant (*p *> 0.05) effect on the expression of COL1A2 after 14 days cell aggregate culture under normal oxygen condition, but under hypoxia culture conditions, the expression of COL1A2 was significantly increased (1.5-fold; Figure 3). Furthermore, the net effect of FGF2 expansion in cell aggregate cultures was to significantly increase (*p *< 0.001) the expression of COL2A1 (200-fold at normal oxygen tension, and 445-fold under hypoxic culture conditions; Figure 3).

The combination of FGF2 expansion and hypoxia therefore increased the capacity of meniscus cells to re-express the matrix-forming phenotype of meniscus cells. Comparison of these expression levels with cells in tissue showed that COL1A2 expression in FGF2-derived cell aggregates under low oxygen was 5-fold less than its expression in tissue, while in non-FGF2-derived cell aggregates under low oxygen COL1A2 expression was decreased 10-fold relative to its expression in tissue. In addition, FGF2 expansion increased the expression of COL2A1 in cell aggregates under low oxygen 5-fold relative to COL2A1 expression in tissue (Figure 3). However, in view of these changes in COL2A1 expression, it was surprising that the expression of SOX9 in tissue was 7-fold that in cell aggregates formed from FGF2-expanded cells (Figures 2 and 3).

COL3A1, which is not expressed in normal cartilage but is expressed in meniscus, was expressed similarly in cell aggregates derived from FGF2-expanded and non-FGF2-expanded cells regardless of oxygen tension, and the expression of COL3A1 in tissue and in the cell aggregates were at similar levels (Figure 3).

**Figure 4:**
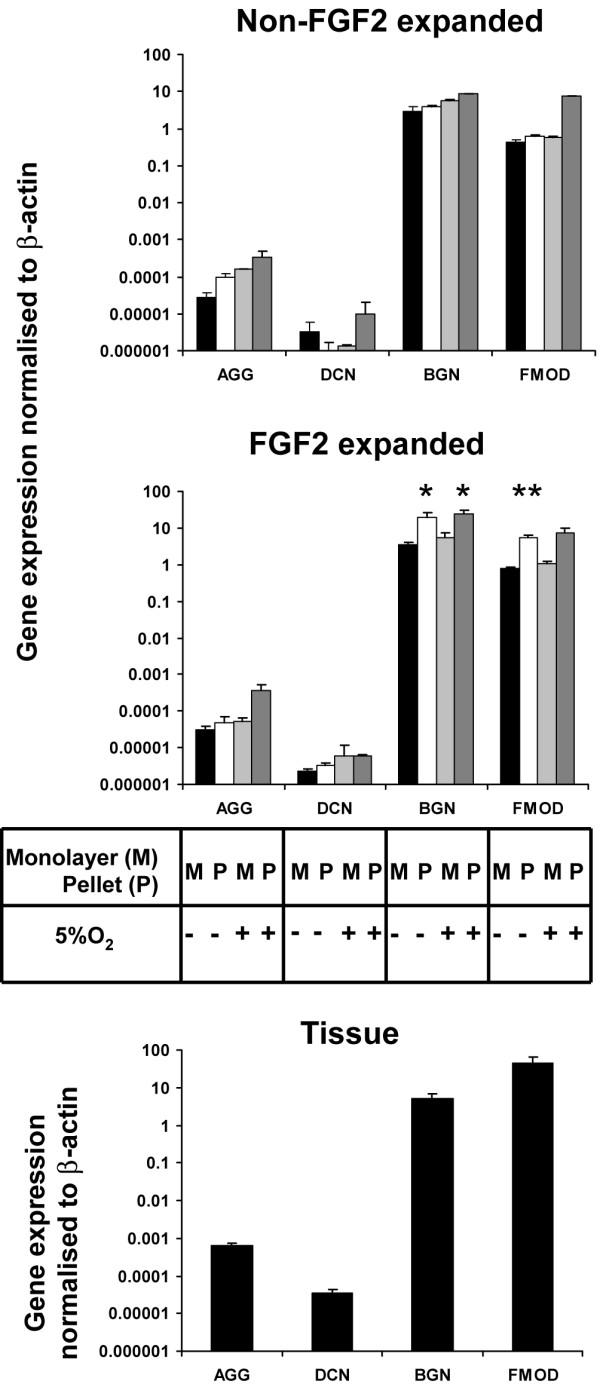
Proteoglycan gene expression in monolayer and aggregate cultures from non-FGF2 and FGF2 expanded meniscus cells. Gene expression levels for monolayer cells (black bars) between P2 and P3 in standard medium (*n *= 3) and for cell aggregate (white open bars) in chondrogenic differentiation medium (*n *= 3) under normal oxygen after 14 days in culture. Gene expression for monolayer cells (light grey bars) between P2 and P3 in standard medium (*n *= 3) and for cell aggregate (dark grey bars) in chondrogenic differentiation medium (*n *= 3) under hypoxia after 14 days in culture. AGG, aggrecan; BGN, biglycan; DCN, decorin; FGF2, fibroblast growth factor 2; FMOD, fibromodulin; P2, passage 2; P3, passage 3.

To characterise the effect of FGF2 expansion on meniscus cells further, we investigated the gene expression of L-SOX5 and SOX6 and also proteoglycans known to be expressed in cartilage and meniscus. L-SOX5, SOX6 and SOX9 cooperatively activate the expression of COL2A1 [28]. There was no change in the expression of L-SOX5 and SOX6 in cell aggregates derived from FGF2-expanded meniscus cells (Figure 2). Aggrecan is the predominant proteoglycan of cartilage and inner meniscus, and decorin, biglycan and fibromodulin are small leucine-rich proteoglycans found within the two tissues. However, the concentration of proteoglycans in meniscal tissue (measured as total glycosaminoglycan (GAG)) is only 12% of the concentration found in cartilage [10,29-31]. Gene expression analysis of 14-day cell aggregates showed that the cell aggregates from FGF2 expansion (Figure 4) had significantly (*p *< 0.05) higher expression of biglycan (6-fold) and fibromodulin (8-fold) (Figure 4). However, at low oxygen tension only biglycan showed a further increase (3-fold) in expression. The expression of biglycan was 5-fold higher in cell aggregates formed from FGF2-expanded cells at low oxygen tension than in tissue (Figure 4), whereas the expression of fibromodulin in tissue was 6-fold that in low-oxygen cell aggregates (Figure 4). There were no significant changes in the mRNA expression of aggrecan but it was one-half that in tissue (Figure 4). The expression of decorin was low and remained unchanged between cell aggregates formed from FGF2-expanded and non-FGF2-expanded cells, but the expression of decorin was 3-fold to 6-fold lower in cell aggregates than that in tissue (Figure 4).

### Effect of FGF2 expansion on matrix formation and proteoglycan matrix deposition

**Figure 5:**
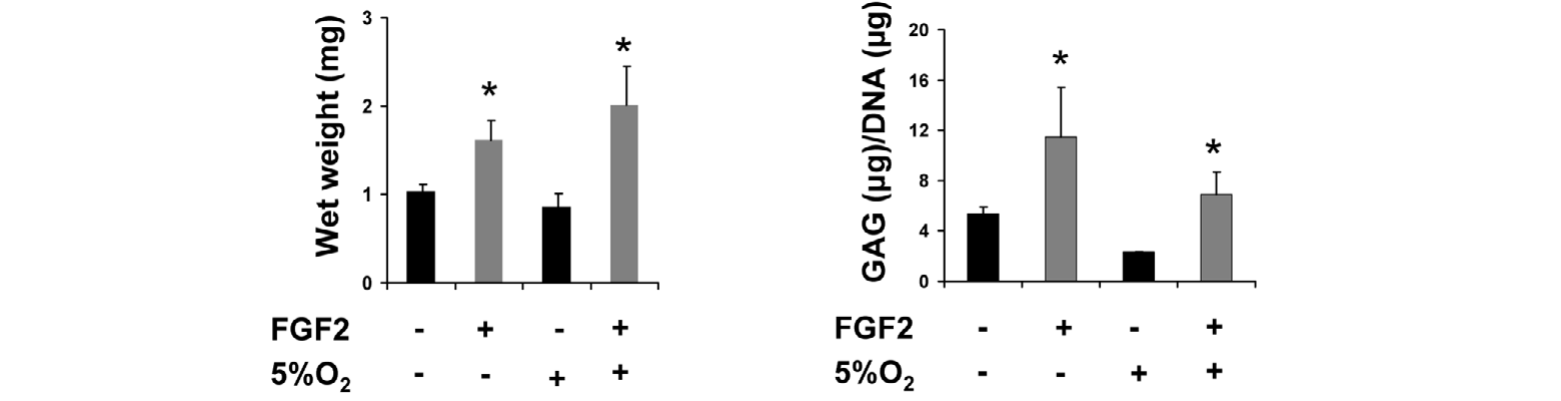
Weights and GAG per DNA content of cell aggregates. Wet weights of cell aggregate derived from non-FGF2-expanded (black bars) and FGF2-expanded (grey bars) meniscus cells under normoxia (-) or hypoxia (+), and GAG content of cell aggregate normalised to DNA content. FGF2, fibroblast growth factor 2; GAG, glycosaminoglycan.

Expanded meniscus cells were cultured in cell aggregates under conditions known to favour chondrocyte matrix assembly. As a measure of the growth and accumulation of ECM in the cell aggregate cultures, the wet weights were recorded after 14 days of culture. The weight of cell aggregates derived from FGF2-expanded cells was significantly (*p *< 0.05) higher (1.5-fold to 2-fold) than that of cell aggregates derived from cells expanded in the absence of FGF2 regardless of oxygen tension (Figure 5). Further analysis of GAG production per cell showed that there was also no significant (*p *> 0.05) effect of low-oxygen culture (Figure 5). However, the accumulation of GAG was 215 to 255% higher in cell aggregate cultures from FGF2-expanded cells than in those from non-FGF2-expanded cells.

**Figure 6:**
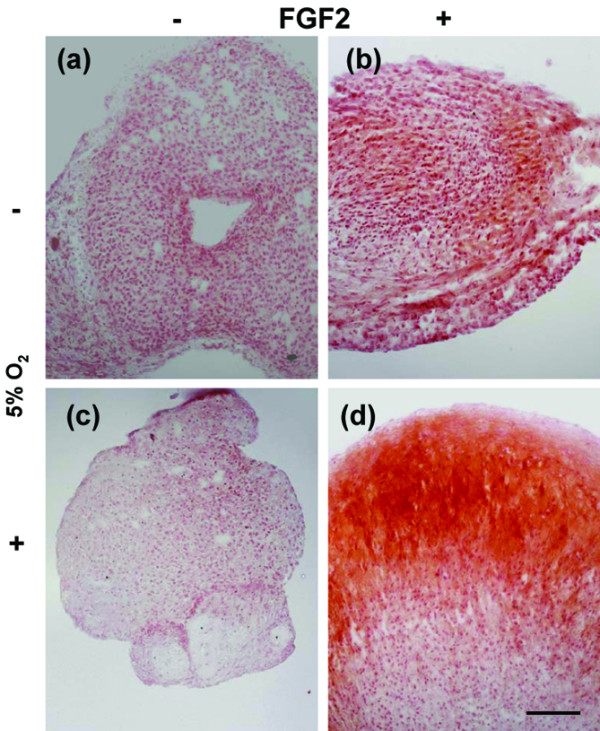
Safranin-O staining of cell aggregates. Histological analysis and matrix accumulation of cell aggregates derived from meniscus cells expanded in the absence (**(a) **normoxia; **(c) **hypoxia) and presence (**(b) **normoxia; **(d) **hypoxia) of fibroblast growth factor 2 (FGF2). Scale bar, 100 μm. FGF2, fibroblast growth factor 2.

Histochemical staining of the cell aggregates with safranin-O was used to assess proteoglycan accumulation. Cell aggregate derived from FGF2-expanded cells under normoxic conditions stained weakly with safranin-O, but the staining was not uniformly distributed (Figure 6b). In contrast, at low oxygen tension there was a strongly stained ECM, particularly in the periphery of the aggregates (Figure 6d). In addition, the cells in the region of strong safranin-O staining had a more rounded morphology, reminiscent of chondrocytes in cartilage (Figure 6d), but staining in the central zone of the cell aggregate was weak. Cell aggregates from non-FGF2-expanded meniscus cells showed no staining with safranin-O regardless of oxygen tension (Figure 6a,c).

### Effect of FGF2 expansion on collagen deposition

**Figure 7:**
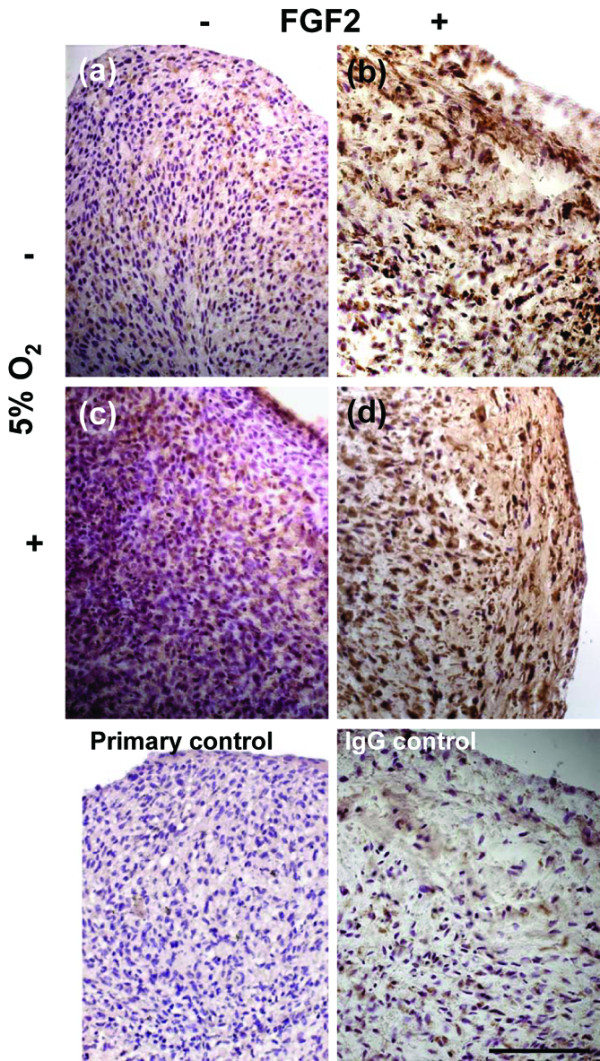
Immunolocalisation of type I collagen in cell aggregates after 14 days of culture. Cell aggregates derived from meniscus cells expanded in the absence (**(a) **normoxia; **(c) **hypoxia) and presence (**(b) **normoxia; **(d) **hypoxia) of fibroblast growth factor 2 (FGF2). No primary antibody control and non-specific IgG control. Scale bar, 100 μm.

**Figure 8:**
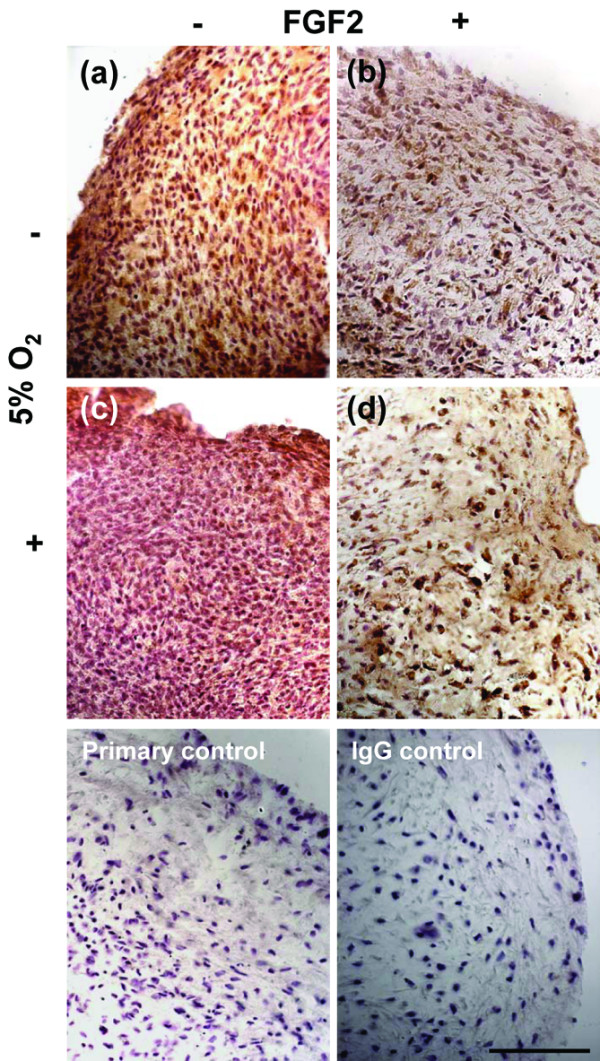
Immunolocalisation of type II collagen in cell aggregates after 14 days of culture. Cell aggregates derived from meniscus cells expanded in the absence (**(a) **normoxia; **(c) **hypoxia) and presence (**(b) **normoxia; **(d) **hypoxia) of fibroblast growth factor 2 (FGF2). No primary antibody control on cell aggregates derived from non-FGF2-expanded and FGF2-expanded cells after culture under hypoxia. Scale bar, 100 μm.

To assess collagen matrix deposition, the cell aggregate cultures were immunostained with antibodies against type II collagen and against type I collagen. The FGF2-expanded cells (Figure 7b) stained less with anti-collagen type I than cell aggregates formed from non-FGF2-expanded cells under normal oxygen tension (Figure 7a,c); under low oxygen tension both FGF2-expanded cells (Figure 7d) and non-FGF2-expanded cells (Figure 7c) stained weakly with anti-collagen type I. Cell aggregates from FGF2-expanded cells (Figure 8b,d) stained strongly for anti-collagen II at 14 days in both normoxic and low-oxygen cultures. In contrast, little or no anti-collagen type II staining was observed in cell aggregate from non-FGF2-expanded cells (Figure 8a,c).

## Discussion

Cell-based strategies to engineer a meniscus substitute has been suggested as an approach to the treatment of meniscal defects. However, attempts to expand human meniscus cells in monolayer culture have resulted in decreased gene expression of ECM components of importance in meniscus function, such as type II collagen [7], which is located mostly in the inner region of the tissue and is thought to endow properties suitable for compressive load distribution [13]. In this study we have investigated the combination of culture under conditions of low oxygen tension and FGF2-stimulated cell expansion as a strategy to augment the re-expression of type II collagen and a matrix-forming phenotype in human meniscus cells. Human meniscus cells showed a chondrogenic response (increased collagen II gene and protein expression) when cultured in cell aggregates regardless of FGF2 presence or absence during monolayer expansion (Figure 3). However, the response was much greater in cell aggregate cultures derived from FGF2-expanded cells (Figure 3). The type II collagen protein was notably more localized in the matrix at the periphery of the cell aggregates and more pericellularly at the central region of the cell aggregates (Figure 8). The chondrogenic response was further enhanced by low oxygen tension, which caused increased gene expression of SOX9. However, the expression of L-SOX5 and SOX6 remained unchanged and low. This was surprising because L-SOX5 and SOX6 interact cooperatively with SOX9 to promote the expression of cartilage-specific genes (such as those encoding COL2A1 and aggrecan) [28]. The enhanced chondrogenic response at low oxygen tension may involve the transcriptional activity of HIF-1α, (hypoxia inducible factor) which modulates the expression of a variety of hypoxia-inducible genes under low oxygen tension [32]. It has been reported that hypoxia promotes the differentiation of murine mesenchymal stroma cells along a chondrocyte pathway in part by activating SOX9 via a HIF-1α-dependent mechanism [33]. Furthermore, HIF-1α has been shown to bind to cAMP-response element-binding protein (CREB)-binding protein (CBP)/p300 [34], which SOX9 uses to exert its cartilage-specific type II collagen gene promoter activity [35]. It was noticeable that in monolayer there was no significant chondrogenic response in changing from normal oxygen tension (20%) to low oxygen tension (5%) compared with changing from monolayer to aggregate (Figure 3). In the comparison between the expression of cells in aggregates and in monolayer, the 3D structure of a cell aggregate, together with oxygen consumption by the cells, would result in a lower oxygen tension within the aggregate than in cells in a monolayer. However, because cell aggregates showed a strong chondrogenic response at 5% and 20% oxygen, any small difference in oxygen tension was clearly not a major factor driving the chondrogenic response.

It was notable that the high gene expression of COL1A2 in cell aggregates formed from FGF2-expanded cells was not correlated with the matrix immunostaining, which was weak with anti-type I collagen. This suggested that there is a more complex control of type I collagen translation, fibrillogenesis and matrix deposition.

Further characterization of the chondrogenic response by human meniscus cells was by gene expression analysis of proteoglycan common to cartilage and meniscus. Aggrecan gene expression was low in meniscal cells and was not influenced by FGF2-mediated cell expansion, but its expression increased in cell aggregate cultures. FGF2-expanded cells expressed higher levels of biglycan and fibromodulin in cell aggregates, and this was unaffected by low oxygen. In non-FGF2-expanded cells, biglycan and fibromodulin expression was similar in monolayer and cell aggregates, but biglycan was increased by low oxygen tension in cell aggregates formed from FGF2-expanded cells. Histology showed an increase in safranin-O staining in cell aggregates formed from FGF2-expanded cells at low oxygen tension. Although this did not reflect a significant statistical increase in GAG/DNA ratio under low-oxygen conditions, the cell aggregates formed were of higher wet weight and this might correspond to a greater increase in cell number.

This study showed that P2 meniscus cells after growth stimulation with FGF2 were able to re-express type II collagen and proteoglycans at both the gene and protein levels. Furthermore, this ability was enhanced by 5% oxygen culture conditions and was higher than with meniscus cells expanded in the absence of FGF2. The cells used in this study were from all regions of the meniscus and thus include cells from the inner avascular region, which contains more collagen type II than the outer vascular region. FGF2 may favour the selective proliferation of the cells from this region and thus sustain the population of meniscus cells with chondrogenic potential. Expansion with FGF2 has been reported to increase the chondrogenic potential of human bone marrow stromal cells [36]. Previous studies by Nakata and colleagues [7] have reported three distinguishable cell types within the human meniscus tissue: small round chondrocyte-like cells, elongated fibroblast-like cells and polygonal cells; they related the loss of collagen II expression in meniscus cells during monolayer expansion with the gradual loss of both the chondrocyte-like and polygonal cell populations to leave predominantly fibroblast-like cells. The mechanism by which FGF2 conferred this ability to re-express type II collagen and proteoglycan in meniscus cells is therefore either by the selective proliferation of chondrogenic cells within the culture or by maintaining the cells in a more plastic and responsive state to chondrogenic stimuli [16].

## Conclusion

We have shown that the loss of collagen II expression after monolayer expansion of human meniscus cells can be circumvented by adding FGF2 during the monolayer expansion phase. Furthermore, the ability of FGF2-expanded meniscus cells to re-express a matrix rich in collagen I and II is enhanced by hypoxia. This combination strategy may improve cell-based approaches to generate the biomechanical properties of meniscus substitutes.

## Abbreviations

3D = three-dimensional; COL = collagen; DMEM = Dulbecco's modified Eagle's medium; ECM = extracellular matrix; FCS = fetal calf serum; FGF2 = fibroblast growth factor 2; GAG = glycosaminoglycan; HIF = hypoxia inducible factor; HMG = high-mobility group; L-SOX5 = Sry-related HMG box-5 (long form); SOX6 = Sry-related HMG box-6; SOX9 = Sry-related HMG box-9.

## Competing interests

The authors declare that they have no competing interests.

## Authors' contributions

ABA conceived, designed and performed the experiments described in this study and was responsible for the initial versions of this manuscript. LMG performed all the immunohistochemical experiments included in this manuscript. WSK was responsible for tissue procurement and processing. TEH supervised and oversaw the completion of the studies as well as the writing of this manuscript. All authors read and approved the final manuscript.
